# Sensing how to balance

**DOI:** 10.7554/eLife.46973

**Published:** 2019-04-17

**Authors:** Fabrice Ango, Raphaël Dos Reis

**Affiliations:** Department of Neuroscience, Institut de Génomique FonctionnelleUniversité de Montpellier, CNRS and INSERMMontpellierFrance

**Keywords:** vestibular, cerebellum, mossy fiber, optogenetics, granule cell, unipolar brush cell, Mouse

## Abstract

How does the inner ear communicate with the cerebellar cortex to maintain balance and posture?

**Related research article** Balmer TS, Trussell LO. 2019. Selective targeting of unipolar brush cell subtypes by cerebellar mossy fibers. *eLife* **8**:e44964. doi: 10.7554/eLife.44964

Keeping your head upright may seem like a trivial task, but the neural circuitry required to perform this task is rather complex and not fully understood. This circuitry starts with the vestibular system: a sensory system in the inner ear that relies on hair cells to detect movements, and to provide our sense of balance and spatial awareness. The vestibular system contains five organs that are sensitive to different types of movement. The sacculus and the utricle detect gravity and linear movements, respectively, and there are three semi-circular canals that detect rotation. Information about these movements is sent from the vestibular system to the cerebellum, which co-ordinates the motor movements needed to maintain posture and balance (Ito, 2006).

The hair cells in the vestibular system contact VG (vestibular ganglion) neurons, which then send sensory information along nerve cells called mossy fibers to the vestibular region of the cerebellum (Dow, 1936). The fibers that send signals directly to the cerebellum are called primary afferents, and the fibers that send signals indirectly via the brainstem nuclei (which also receive information from other sensory systems) are called secondary afferents (Maklad and Fritzsch, 2003; see Figure 1).

**Figure 1.**
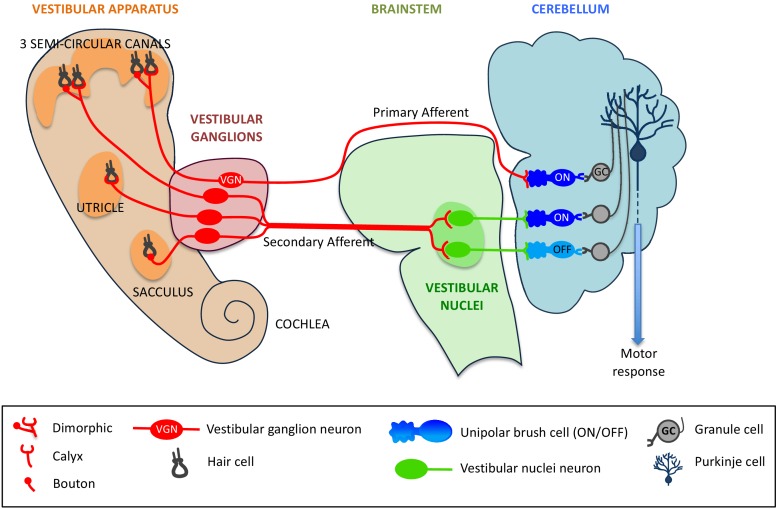
Primary and secondary afferents from the vestibular system to the cerebellum. Neurons from the hair cells (black) within the five organs of the vestibular system (left) form different types of synapses – dimorphic, calyx or bouton – with vestibular ganglion (VG) neurons (red). Mossy fibers (also in red) can project directly from the VG neurons to the cerebellum (in which case they are called primary afferents), or indirectly via vestibular nuclei within the brainstem (secondary afferents). The primary afferents (red) form synapses with a type of unipolar brush cell (UBC) called an ON UBC, whereas secondary afferents form synapses with both ON UBCs (dark blue) and OFF UBCs (light blue). UBCs form synapses with granule cells (grey), which in turn make contact with Purkinje cells (dark blue), which convey motor responses to the rest of the body.

Both the primary and secondary afferents form synapses with neurons called granule cells in the cerebellum: granule cells are the most numerous excitatory neurons in the brain (Chadderton et al., 2004). A single mossy fiber can activate hundreds of granule cells which, in turn, form synapses with the dendrites of Purkinje cells. These cells are the sole output neurons from the cerebellar cortex and they have a crucial role in motor learning.

However, this is not the full story because the vestibular region of the cerebellum also contains a high proportion of excitatory neurons called unipolar brush cells (UBCs). These cells, which receive input from just a single mossy fiber, form synapses with the granule cells (Mugnaini et al., 2011). UBCs essentially create an intermediate step in the circuitry, where signals sent between mossy fibers and granule cells can be modified. How the signal is modified depends on the type of UBC involved: ON UBCs will have an amplified response, whereas OFF UBCs will have a dampened response (Borges-Merjane and Trussell, 2015). However, there is much about the pathways connecting the vestibular system and cerebellum that is not fully understood: for instance, how is information from the vestibular system processed once it reaches the cerebellum? Now, in eLife, Timothy Balmer and Laurence Trussell of Oregon Health and Science University report the results from experiments on genetically-modified mice that will help to answer such questions (Balmer and Trussell, 2019).

The two researchers used a combination of transgenic mice and retrograde-infecting viruses to map the morphology of the VG neurons. These experiments showed that the primary afferents largely originated at the three semi-circular canals of the vestibular system, and that the dendrites of the VG neurons mostly had a dimorphic morphology (see Figure 1). These results, combined with our current knowledge of the sensory organs of the vestibular system, led Balmer and Trussell to conclude that the primary afferents are responsible for sensing rotational movements of the head (Fernández et al., 1988).

An optogenetic approach was then employed to assess which neurons in the cerebellum were targeted by these dimorphic VG neurons. Using light to stimulate light-sensitive ion channels in VG neurons led to electric impulses being observed in UBCs in the cerebellum. The characteristics of this response were distinctive of ON UBCs, and a response could not be detected from the OFF UBCs. This finding was further bolstered by immunohistochemical staining, which showed primary afferent synapses projecting solely onto the ON UBC subtype. These data suggest that direct projections of VG neurons solely target ON UBCs, but not OFF UBCs.

Finally, Balmer and Trussell investigated the differences between the direct and the indirect pathways by expressing a light-sensitive channel in the vestibular region of the brainstem. In contrast with primary afferents, secondary afferents targeted both ON and OFF UBCs to a similar degree (see Figure 1).

The complexity of the circuitry revealed by Balmer and Trussell seems suited to the delicate task of balancing one's head, but a number of questions remain. In particular, how and where do the primary and secondary afferent pathways converge to trigger the relevant responses? An interesting follow up to this study would be to compare the role played by UBCs in maintaining balance and posture with their role in processing the other types of sensory inputs that are sent to the cerebellum.
